# Curcumin and Its Supramolecular Complex with Disodium Glycyrrhizinate as Potential Drugs for the Liver Fluke Infection Caused by *Opisthorchis felineus*

**DOI:** 10.3390/pathogens12060819

**Published:** 2023-06-09

**Authors:** Maria N. Lvova, Denis V. Ponomarev, Alena A. Tarasenko, Anna V. Kovner, Galina A. Minkova, Michail A. Tsyganov, Meijun Li, Yan Lou, Veronica I. Evseenko, Alexander V. Dushkin, Irina V. Sorokina, Tatiana G. Tolstikova, Viatcheslav A. Mordvinov, Damira F. Avgustinovich

**Affiliations:** 1 Institute of Cytology and Genetics, Siberian Branch of Russian Academy of Sciences, 630090 Novosibirsk, Russia; ponomarevd@bionet.nsc.ru (D.V.P.); tarasenkoaa@bionet.nsc.ru (A.A.T.); kovner@bionet.nsc.ru (A.V.K.); maksimova@bionet.nsc.ru (G.A.M.); tsyganov@bionet.nsc.ru (M.A.T.); mordvin@bionet.nsc.ru (V.A.M.); 2 Department of Natural Sciences, Novosibirsk State University, 630090 Novosibirsk, Russia; 3 Institute of Solid State Chemistry and Mechanochemistry, Siberian Branch of Russian Academy of Sciences, 630090 Novosibirsk, Russia; evseenkov@inbox.ru (V.I.E.); dushkin@solid.nsc.ru (A.V.D.); 4 N.N. Vorozhtsov Novosibirsk Institute of Organic Chemistry, Siberian Branch of Russian Academy of Sciences, 630090 Novosibirsk, Russia; sorokina.irina55@gmail.com (I.V.S.); tg_tolstikova@mail.ru (T.G.T.)

**Keywords:** *Opisthorchis felineus*, hamster, curcumin, supramolecular complex of curcumin, praziquantel, liver inflammation, gene expression

## Abstract

Opisthorchiosis is a parasitic liver disease found in mammals that is widespread throughout the world and causes systemic inflammation. Praziquantel remains the drug of choice for the treatment of opisthorchiosis, despite its many adverse effects. An anthelmintic effect is attributed to the main curcuminoid of *Curcuma longa* L. roots—curcumin (Cur)—along with many other therapeutic properties. To overcome the poor solubility of curcumin in water, a micellar complex of curcumin with the disodium salt of glycyrrhizic acid (Cur:Na_2_GA, molar ratio 1:1) was prepared via solid-phase mechanical processing. In vitro experiments revealed a noticeable immobilizing effect of curcumin and of Cur:Na_2_GA on mature and juvenile *Opisthorchis felineus* individuals. In vivo experiments showed that curcumin (50 mg/kg) had an anthelmintic effect after 30 days of administration to *O. felineus*-infected hamsters, but the effect was weaker than that of a single administration of praziquantel (400 mg/kg). Cur:Na_2_GA (50 mg/kg, 30 days), which contains less free curcumin, did not exert this action. The complex, just as free curcumin or better, activated the expression of bile acid synthesis genes (*Cyp7A1*, *Fxr*, and *Rxra*), which was suppressed by *O. felineus* infection and by praziquantel. Curcumin reduced the rate of inflammatory infiltration, whereas Cur:Na_2_GA reduced periductal fibrosis. Immunohistochemically, a decrease in liver inflammation markers was found, which is determined by calculating the numbers of tumor-necrosis-factor-positive cells during the curcumin treatment and of kynurenine-3-monooxygenase-positive cells during the Cur:Na_2_GA treatment. A biochemical blood test revealed a normalizing effect of Cur:Na_2_GA (comparable to that of curcumin) on lipid metabolism. We believe that the further development and investigation of therapeutics based on curcuminoids in relation *Opisthorchis felineus* and other trematode infections will be useful for clinical practice and veterinary medicine.

## 1. Introduction

The infectious disease, opisthorchiosis (the name of the pathology is given in accordance with the recommendation of the World Association for the Advancement of Veterinary Parasitology) [1], develops in fish-eating mammals when one of two species of helminths of the Opisthorchiidae family gets into the liver of the host: either *Opisthorchis felineus* or *O. viverrini*. They cause hepatobiliary diseases, including hepatomegaly, cholangitis, cholecystitis, periductal and periportal fibrosis, gallstones, and even cholangiocarcinoma [2,3]. Opisthorchiosis caused by *O. viverrini* is endemic to Thailand, Lao PDR, Cambodia, and Vietnam [4], whereas opisthorchiosis induced by *O. felineus* is endemic to western Siberia (mostly in the Ob–Irtysh basin) and occurs in Eastern Europe (e.g., Ukraine, Belorussia, and Baltic countries) and in Western Europe (e.g., Germany and Italy) [2,5,6,7]. In south-eastern Asia, more than 90 million people are at risk of infection, and >12 million people are estimated to be infected with *O. viverrini* [4]. Opisthorchiosis caused by *O. felineus* has been diagnosed in 1.6 million people, and another 12.5 million are at risk of the infection [6].

Praziquantel (PZQ) is the drug of choice recommended by the WHO for the treatment of infection caused by *O. felineus* or *O. viverrini* [8]. It is a broad-spectrum anthelmintic drug that affects not only trematodes (*Opisthorchis* spp., *Clonorchis sinensis*, *Fasciola hepatica*, and *Schistosoma* spp.), but also cestodes. PZQ has some adverse effects (such as headaches, vertigo, nausea, and fatigue) [9] and is not always 100% effective against the infection caused by *O. viverrini* [10] or *O. felineus* [11,12], whereas repeated PZQ treatments are associated with an elevated risk of cholangiocarcinoma [13]. Therefore, there is a constant search for new anthelmintic drugs, including those that originate from plants.

In this regard, curcumin (Cur), a hydrophobic polyphenol from *Curcuma longa* L., deserves attention. Due to its antioxidant, anti-inflammatory, analgesic, antimicrobial, antiviral, and antitumor properties, Cur exerts a significant beneficial action in various pathological conditions, including inflammatory diseases and cancers [14,15]. Its ability to penetrate the blood–brain barrier allows for the use of Cur in diseases of the central nervous system [14,16]. It has been experimentally shown in golden hamsters (*Mesocricetus auratus*) that Cur protects against oxidative/nitrative stress and liver injuries induced by *O. viverrini* infection and by the PZQ treatment [17,18]. Its antifibrotic effect appears after 3 months of its consumption with food by *O. viverrini*-infected golden hamsters [19]. No anthelmintic effect of Cur on *O. viverrini* has been found, but this activity has been indirectly investigated by means of the number of eggs per gram of feces [17]. Nonetheless, the significant efficacy of Cur against several cestodes and nematodes has been reported [20,21,22].

On the other hand, Cur also has substantial disadvantages: poor bioavailability due to low water solubility and reduced absorption in the gastrointestinal tract after oral administration, poor stability in body fluids, a high rate of metabolism, and rapid systemic elimination [23]. In this regard, numerous methods and approaches are being devised to improve the bioavailability of Cur. For example, a nano-based drug delivery system has improved not only the water solubility and bioavailability of Cur, but also its chemical stability, thereby preventing its enzymatic- and pH-induced degradation and increasing its circulation within the body [24]. In addition, a method for obtaining Cur nanoparticles can be used to overcome the toxicity that is seen when high doses of the pure substance are used [25]. An advantage of Cur nanoencapsulation has been demonstrated in *O. viverrini*-infected golden hamsters: when combined with PZQ, it more effectively reduces periductal fibrosis, improves the bile canaliculi morphology, and upregulates several genes involved in the bile acid metabolism [26].

Our studies involve the mechanochemical method of complexing Cur with water-soluble polymers in VM-1 ball mills [27]. Complexes with the disodium salt of glycyrrhizic acid (Na_2_GA) created using this method, have a good water solubility property, penetration capacity, and therefore, bioavailability [28,29]. It is known that glycyrrhizic acid (GA) is a highly soluble plant saponin that can become integrated with the lipid bilayer and enhance membrane fluidity and permeability [30]. It is reported that GA can form noncovalent intermolecular complexes with drugs or include drug molecules into supramolecular structures (micelles), thus making it possible to increase water solubility of the compounds and to improve their bioavailability. We have previously found that a water-soluble complex of PZQ with Na_2_GA in a 1:10 weight ratio allows the PZQ dose to be decreased 11-fold for the effective killing of *O. felineus* [12], and this advancement is important for reducing the side effects of medications.

In the present study, we used Cur and its complex with Na_2_GA at a 1:1 molar ratio (Cur:Na_2_GA), which was administered orally for 30 days to golden hamsters infected with *O. felineus* helminths. The aim of the study was to determine their anthelmintic activity in vitro and in vivo, as well as to assess the state of the parasite’s host according to molecular, histological, and behavioral parameters.

## 2. Materials and Methods

### 2.1. Animals

We used golden hamster males (*Mesocricetus auratus*) aged 2.5–3.0 months obtained from the SPF vivarium at the Institute of Cytology and Genetics, the Siberian Branch of the Russian Academy of Sciences (ICG SB RAS). The animals were kept on a light: dark cycle of 12:12 h, at an air temperature of 23–24 °C, with sufficient amounts of feed pellets and water. Hamsters were housed at one per cage (measuring 36 cm × 23 cm × 12 cm), in which wood blocks made of leaved trees were placed, which are necessary for these animals to grind their teeth. All procedures were carried out in accordance with directives of the European Communities Council, dated 24 November 1986 (86/609/EEC), and a decision by the Bioethical Committee of the ICG SB RAS (protocol no. 39, as of 27 September 2017).

### 2.2. Drugs

Samples of the Cur complex with Na_2_GA (Cur:Na_2_GA, molar ratio 1:1 or weight ratio 6.56 g of Cur per 15.44 g of Na_2_GA) were prepared and initially physicochemically characterized as described earlier [31]. The water solubility of Cur within the Cur:Na_2_GA complex was more than 400-fold higher, which increased the bioavailability by approximately 19-fold. For the in vitro experiments, pure PZQ powder (Sigma–Aldrich (Shanghai) Trading Co., Ltd., Shanghai, China) and Cur (St. Louis, MO, USA) were dissolved in 0.5% dimethyl sulfoxide (DMSO; Sigma-Aldrich, St. Louis, MO, USA); Cur:Na_2_GA and Na_2_GA, which was purchased from Shaanxi Sciphar Biotechnology Co., Ltd. (Xi’an, China, purity: 98%), were dissolved in distilled water. For the in vivo experiments, PZQ and Cur were dissolved in 10% Tween 80, whereas Cur:Na_2_GA was dissolved in water.

### 2.3. Design of In Vitro Experiments

The anthelmintic properties of PZQ, Cur, Na_2_GA, and of the complex Cur:Na_2_GA (1:1) were evaluated as their ability to inhibit the motility (a) of adult *O. felineus* liver flukes isolated from liver bile ducts of hamsters infected via the intragastric administration of 100 metacercaria per hamster or (b) of *O. felineus* individuals obtained via the stimulation of excystation of metacercariae, termed newly excysted metacercariae (NEMs), collected from naturally infected fish (*Leuciscus idus*) caught in the Ob River near the city of Novosibirsk (Western Siberia). Briefly, metacercariae were thoroughly washed with sterile phosphate-buffered saline containing antibiotics (100 μg/mL penicillin and 100 μg/mL streptomycin). Metacercariae were transferred to a culture plate, 0.06% trypsin solution with 0.03% EDTA in Dulbecco’s buffer was added. The plate was placed in a CO_2_ incubator for 15–30 min to stimulate the release of larvae from cysts. Then, after washing the newly excysted worms 5 times with the incubation medium, the analyzed substances were added to the wells.

Testing was performed on live, intact parasites in culture plate wells containing the RPMI 1640 incubation medium (Life Technologies, Carlsbad, CA, USA) supplemented with 100 U/mL penicillin, 100 mg/mL streptomycin, 25 μg/mL amphotericin B, and 1% glucose. Tested substances or appropriate solvents (0.5% DMSO or water) were added to the wells of a culture plate containing either NEMs or mature worms; the culture plates were then kept for 24 h in a CO_2_ incubator (37 °C and 5% CO_2_). The concentration range for all substances was 0.1–1000 μg/mL, except for PZQ (0.1–100 μg/mL). For each concentration of each drug, 6–10 adult liver flukes or 40–100 NEMs of *O. felineus* per well were used, at least in duplicate. Twenty-four hours after the addition of a substance, its effectiveness was evaluated under a microscope as the degree of inhibition of worm motility on a four-point scale: 4, active body movements or the migration of a worm; 3, weak wavelike movements of the whole body; 2, movement of the body only near the sucker; 1, complete immobility [32]. Half-maximal inhibitory concentrations (IC_50_), i.e., effective concentrations causing the immobility of 50% of the individuals, were calculated using Compu Syn software [33].

### 2.4. Design of In Vivo Experiments

**Figure 1:**
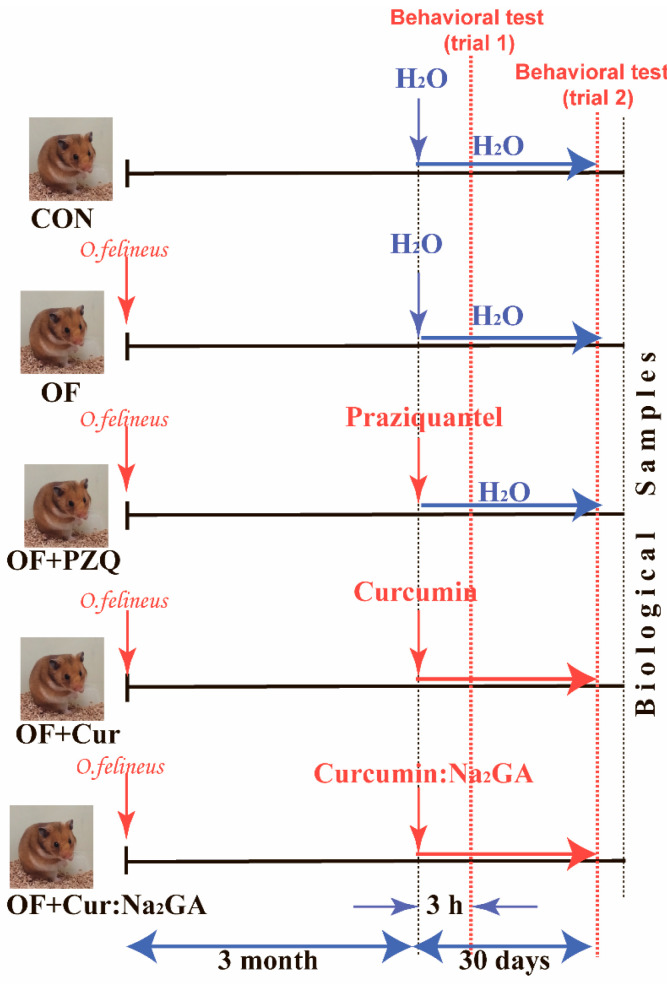
Design of the experiment.

Two-month-old hamsters were intragastrically infected with metacercariae of *O. felineus* (100 larvae per hamster) with probes (Braintree Scientific, Inc., Braintree, MA, USA) [34]. After 3 months, the infected animals were distributed into several groups (6–8 in each), and the following substances were intragastrically administered by means of special probes (Braintree Scientific, Inc.): (1) 30 days of the added water treatment (group OF); (2) a single administration of PZQ, followed by 30 days of the added water treatment (group OF + PZQ); (3) 30 days of the Cur treatment (group OF + Cur); (4) 30 days of treatment with the Cur:Na_2_GA complex (1:1) (group OF + Cur:Na_2_GA). Additionally, there was a group of non-infected animals (CON hamsters) that were given additional water for 30 days (to mimic chronic stress due to drug administration) (Figure 1).

Doses of PZQ (400 mg/kg) and Cur (50 mg/kg) were chosen according to published data [11,26,35,36] and by taking into account the possible toxicity of Cur when it is chronically administered [25]. The dose of the new Cur:Na_2_GA complex was the same as that of Cur, however, the mass content of Cur in it was 3 times lower [31]. After 3 h and 30 days after the administration of the compound, the behavior of the hamsters from five groups was evaluated in the open field test. Two days after the second trial of the test, all hamsters were killed via rapid decapitation, the livers and spleens were excised, and their weights per gram of body weight of hamsters were calculated. From the left lateral lobe of the livers, a standard small piece was cut out closer to the central bile duct; this sample was placed in liquid nitrogen, and then stored at −70 °C until the analysis of gene expression. The number of adult *O. felineus* liver flukes was determined in the remaining liver. In each group, the livers of some animals (4–5 hamsters) were fixed in 10% buffered formalin for subsequent histopathological and immunohistochemical examination. Blood from each animal was collected into vials, which was centrifuged (4 °C, 20 min, and 3000 rpm) to obtain serum for biochemical analyses.

### 2.5. The Open Field Test

It is known that PZQ [37] and Cur [16,24,38] cross the blood–brain barrier, and therefore, they can affect the central mechanisms of behavior regulation in humans and animals. The plasma concentration of PZQ reaches a maximum after 2.5–3.0 h, and the elimination half-time can be >8–9 h [39]. In this context, adverse events can be observed in humans within 3–24 h [9]. The elimination half-time of orally administered Cur (2 g/kg) in rats is reported to be 1.7 ± 0.5 h [23]. Even at this dose (which is much higher than the one we used), only moderate serum concentrations are achieved during a 4 h period [40]. The chronic administration of Cur is most commonly used, especially for therapeutic purposes [23,41]. Accordingly, the first trial of the behavioral test of the animals was carried out at 3–6 h after the administration of substances, and the second trial was carried out after 30 days.

**Figure 2:**
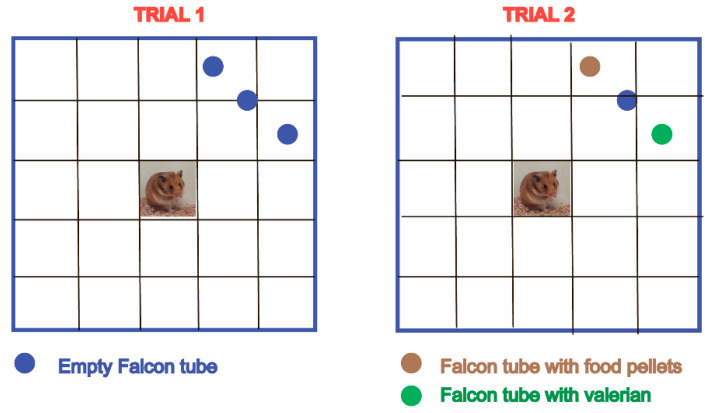
The open field test given to hamsters at 3–6 h after a single administration (Trial 1) and after 30 days (Trial 2) of the administration of substances.

In our experiments, we used an 80 cm × 80 cm field divided by drawn lines into 25 squares (16 cm × 16 cm). In one of corners of the field at a distance of 32 cm–31 cm–32 cm from the center, three round holes with a diameter of 3 cm were cut out, into which Falcon^®^ (Los Angeles, CA, USA) tubes were inserted (Figure 2).

During the first trial of testing of hamsters (3–6 h after the administration of the tested substances), all Falcon^®^ tubes were closed with lids. In the second trial, after 30 days of administration of the substances, a dry preparation made from the roots and rhizomes of valerian (*Valeriana officinalis*) (an unfamiliar smell for hamsters) was placed into one of the non-central Falcon^®^ tubes; into the other one, non-central Falcon^®^ tube feed pellets were placed (a familiar smell for hamsters). These Falcon^®^ tubes had five holes with a diameter of 5 mm. The Falcon^®^ tube between them was closed with a lid (a neutral smell). Five minutes before the testing, each animal was brought to the test room for activation and adaptation to the new conditions. Then, the hamster was placed in the center of the field, and their behavior was recorded for 5 min using a special video camera (GigE Vision standard). After each animal, the field was thoroughly washed and dried off with napkins. Further behavioral analysis was performed in the Behavioral Observation Research Interactive Software (BORIS v.7.9.19) [42]. In both test trials, we recorded the number of crossed squares (locomotor activity), the duration and number of rearings of hamsters (exploratory activity), active digging in corners and approaches to Falcon^®^ tubes containing different contents (selective interest).

### 2.6. Biochemical Assays

The activity of enzymes, alanine aminotransferase (ALT) and aspartate aminotransferase (AST), and concentrations of glucose (GLU), total protein (TP), total cholesterol (CHOL), and triglycerides (TG) in the blood serum of hamsters were determined with standard kits (Biocon, India). The measurements were performed using a Photometer-5010 biochemical semiautomatic analyzer (Boehringer Mannheim, Mannheim, Germany).

### 2.7. RNA Isolation for Real-Time PCR

Total RNA was isolated using the AxyPrep Multisource Total RNA Miniprep Kit (Axygen Biosciences, Union City, CA, USA). Concentrations of RNA were determined using a NanoDrop spectrophotometer (ND1000, NanoDrop Technologies, Wilmington, DE, USA). DNA was eliminated by digestion with DNase I, RNase-free (Thermo Scientific (Waltham, MA, USA)). For cDNA synthesis, we used the Revert Aid First Strand cDNA Synthesis Kit (Thermo Scientific, European Union). Expression levels of genes *Il1b*, *Tnf*, *Cyp7a1*, *Fxr*, *Rxr*, and *Gapdh* were determined by real-time PCR in the presence of EVAGreen (Syntol, Moscow, Russia) using a CFX96 real-time PCR system (Bio-Rad, Hercules, CA, USA), and PCR product specificity was assessed by melting curve analysis. The primer sequences were as follows: interleukin 1 beta *Il1b* F (5′-ACAGAAATGCCTCGTGCTGT-3′) and *Il1b* R (5′-GTGGGCGTGTCACCTTTCAT-3′); tumor necrosis factor, *Tnf* F (5′-GACGGGCTGTACCTGGTTTA-3′) and *Tnf* R (5′-GAGTCGGTCACCTTTCTCCA-3′); cholesterol 7-alpha-monooxygenase, *Cyp7A1* F (5′-CACAAACTCCTTGTCATACC-3′), and *Cyp7A1* R (5′-AGTGAATGCAAAGCATCTCC-3′); farnesoid X receptor, *Fxr* F (5′-CAAGTGACCTCCACGACCAA-3′), and *Fxr* R (5′-CGTGACTGGTAGCCATTTCTG-3′); retinoid X receptor, *Rxr* F (5′-CTCAATGGCGTCCTCAAGGT-3′), and *Rxr* R (5′-ACCGGTTTCTCTGCCTCTTG-3′); glyceraldehyde 3-phosphate dehydrogenase, *Gapdh* F (5′-ATCTCTTCGTGCAGTGCCA-3′), and *Gapdh* R (5′-ACTGGAACATGTAGACCATGTAGT-3′). The amplification efficiencies were 90% to 110% for each primer pair. The *Gapdh* gene was chosen as an endogenous internal control [43]. Triplicate real-time PCR was performed for each sample. A fold change in a target gene’s expression (normalized to the controls) was calculated using the threshold cycle values (C_t_; CFX96 software).

### 2.8. Histological and Immunohistochemical Examination of Liver Sections

To obtain histological sections from all the lobes of hamster livers fixed in 10% formalin (about 7 days), pieces containing bile ducts and parenchyma were cut off, placed in STP-120 automatic histological processing apparatus (Thermo Scientific, Waltham, MA, USA), and embedded in a synthetic paraffin medium (HISTOMIX, Biovitrum, St. Petersburg, Russia) using an EC-350 embedding machine (Thermo Scientific, Waltham, MA, USA). Sections that were 3.5–4 μm thick were prepared using a Microm HM 355S rotary microtome (Thermo Scientific, Waltham, MA, USA). Standard staining techniques were employed: basic overview staining with hematoxylin and eosin and Van Gieson (detecting connective tissue fibers). After staining, the tissue sections were visualized under an Axioskop 2 Plus microscope with an AxioCam MRc camera (Carl Zeiss, Oberkochen, Germany) and AxioVision software (rel. 4.12). During this analysis, these parameters were assessed: inflammatory infiltration, the proliferation of small bile ducts, cholangiofibrosis, and periductal fibrosis. The relative volumetric extent of destructive changes in the liver was evaluated using a closed test system targeting 100 points with an area of 3.64 × 10^5^ μm^2^ and using ImageJ software (https://imagej.nih.gov/ij/) (accessed on 1 December 2022). The scoring method for measurement data has been previously described in detail [44].

To determine the phenotype of effector cells and the degree of their presence in the liver, an indirect biotin-free peroxidase immunohistochemical method was utilized to stain the paraffin sections with an immunohistochemical kit (SpringBioScience Kit HRP-125, Pleasanton, CA, USA) and primary antibodies specific to IL-6 (Abcam, ab6672, 1:100), IL-1β (Abcam, ab9722, 1:100), TNFα (Abcam, ab6671, 1:100), IL-10 (Abcam, ab34843, 1:100), and kynurenine-3-monooxygenase (Cloud-Clone Corp., Katy, TX, USA, DF12118, 1:100). Staining was performed according to the manufacturers’ protocols. The slices were coverslipped using the VitroGel mounting medium (Biovitrum, St. Petersburg, Russia) and visualized under an AxioImager A1 microscope (Zeiss) with an AxioCam MRc camera (Zeiss). At the end, the comparative analysis of IL-6-, IL-1β-, TNF-, IL-10-, and kynurenine-3-monooxygenase-positive staining was carried out among the groups of hamsters.

### 2.9. Statistical Analysis

All results were statistically processed using one-way analysis of variance (ANOVA) in STATISTICA 6.0 software, followed by the *post hoc* comparison of parameters among the groups by Fisher’s least significant difference (LSD) test. Behavioral parameters were processed by the non-parametric Mann–Whitney *U* test. Data are presented as means ± SEM. Differences were considered to be statistically significant at *p* ≤ 0.05 and marginally significant (tendency) at 0.05 ≤ *p* ≤ 0.1.

## 3. Results

### 3.1. In Vitro Data

Data on the half-maximal inhibitory concentration (IC_50_), which causes the inhibition of motility in 50% of newly excysted metacercariae (NEMs) and of adult *O. felineus* liver flukes after incubation with test substances, are presented in Table 1.

In all cases, the sensitivity to these substances was higher among adult *O. felineus* than it was among NEMs. Against the adult worms, the most effective substance was PZQ; the effect of Cur was 2.4 times weaker, and the effect of the Cur:Na_2_GA complex was 14.5 times weaker. Against NEMs, the effectiveness rates of PZQ and Cur were comparable, while the complex was 54 times weaker than PZQ was.

**Figure 3:**
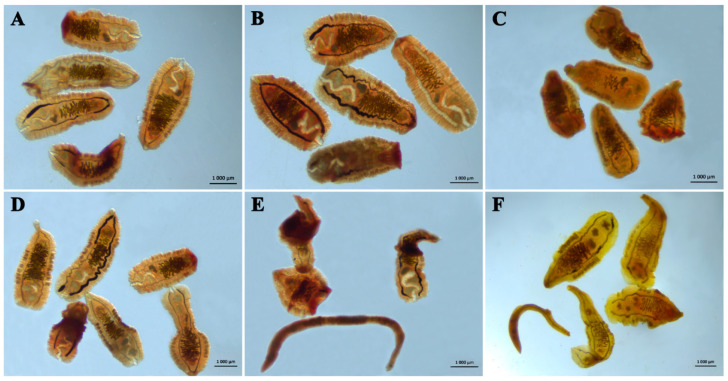
Phenotypes of *O. felineus* liver flukes after 24 h of exposure to the tested substances. Representative photos are shown. (**A**–**C**) These are groups where water was used as a solvent: (**A**) one without a drug; (**B**) one with 1000 μg/mL Na_2_GA; (**C**) one with 1000 μg/mL Cur:Na_2_GA (1:1). (**D**–**F**) These are groups where 0.5% DMSO served as the solvent: (**D**) one without a drug; (**E**) one with 100 μg/mL PZQ; (**F**) one with 1000 μg/mL Cur.

**Figure 4:**
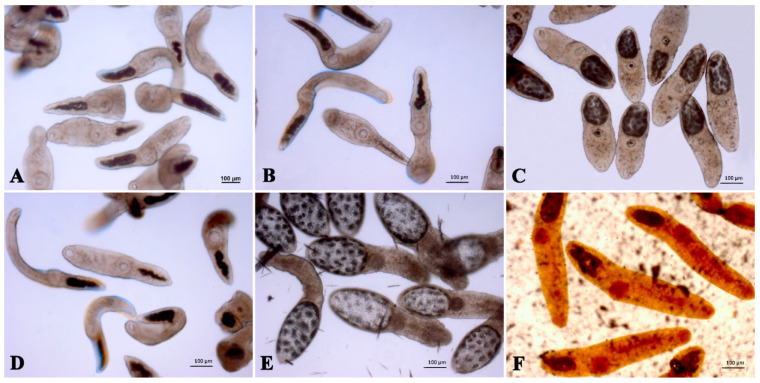
Representative photos of NEMs of *O. felineus* after 24 h of treatment with one of the substances. (**A**–**C**) These are groups where water was used as a solvent: (**A**) one without a drug; (**B**) one with 1000 μg/mL Na_2_GA; (**C**) one with 1000 μg/mL Cur:Na_2_GA (1:1). (**D**–**F**) These are groups where 0.5% DMSO served as the solvent: (**D**) one without a drug; (**E**) one with 100 μg/mL PZQ; (**F**) one with 1000 μg/mL Cur.

The external appearance of adult liver flukes and of NEMs of *O. felineus* at 24 h after incubation with the maximum concentration of tested substances is presented in Figure 3 and Figure 4.

As displayed in Figure 3, the external appearance of mature helminths after incubation with one of the solvents (water: Figure 3A; 0.5% DMSO: Figure 3D) was the same: the worms were transparent and actively moved their whole bodies. After Na_2_GA was added (Figure 3B), all the flukes stayed alive, but their excretory bladder enlarged. After the treatment with Cur:Na_2_GA (Figure 3C), all liver flukes were in an immobilized state, and their bodies were considerably compacted and deformed. The greatest negative effects were exerted by PZQ (Figure 3E) and Cur (Figure 3F). After exposure to PZQ, all helminths were dead, and their bodies were severely deformed due to muscle contraction. Cur also killed the helminths, but there was almost no deformation of the body. In this case, an appreciable yellow staining of the parenchyma was noticed.

Figure 4 shows that the morphological state of NEMs did not differ between the control groups (the addition of water (Figure 4A) or 0.5% DMSO (Figure 4D)): all individuals actively moved, their whole bodies contracted, the parenchyma was transparent, and the external contours were smooth. After exposure to Na_2_GA (Figure 4B), the morphology of metacercariae did not differ from that of the controls, and only 7% of individuals had a low level of mobility without a change in appearance. After the treatment with Cur:Na_2_GA (Figure 4C), 90% of the NEMs were immobile, and 10% were poorly mobile, with occasional convulsive movements. At the same time, all individuals were found to be shortened and had an enlarged excretory bladder. The strongest negative effects were exerted by PZQ (Figure 4E) and Cur (Figure 4F). After PZQ was added, all individuals were dead, and their enlarged bodies had a swollen appearance. At the same time, the parenchyma was gray, and the excretory bladder had inhomogeneous contents and was excessively enlarged. After the treatment with Cur, post-mortem alterations were also noticeable: the bright yellow bodies of NEMs swelled and did not have a clear-cut, organostructural morphology.

The promising in vitro results on the anthelmintic properties of Cur and its complex Cur:Na_2_GA prompted the comparative analysis of the effects of these substances in *O. felineus*-infected laboratory animals.

### 3.2. In Vivo Data

#### 3.2.1. Animals’ Behavior in the Open Field Test

As presented in Table 2, the hamsters in all five groups did not differ in the duration of their orientation reaction when they were placed in the center of the field, either at 3 h after a single administration or after 30 days of the daily administration of substances.

All hamsters had the same reaction to the smell of the food: the time spent near a Falcon^®^ centrifuge tube containing feed pellets was longer than the time spent near an empty Falcon^®^ tube, and the difference was the greatest among the hamsters treated with Cur:Na_2_GA. This result suggested that neither *O. felineus* infection nor the administration of PZQ, Cur, or Cur:Na_2_GA during the course of the infection impaired the hamsters’ sense of smell, and they preferred to be near a Falcon^®^ tube with a familiar food odor. By contrast, the reaction to an unfamiliar smell (valerian) was comparable to the reaction to an empty Falcon^®^ tube in each group, including for the control (CON) hamsters. Consequently, the animals ignored the unfamiliar smell, which also indicated that the hamsters retained a normal sense of smell.

In contrast to CON hamsters, the infected hamsters, including those treated with PZQ or Cur:Na_2_GA, showed markedly less locomotor activity during the second trial of the test, as measured by the number of crossed squares. In addition, in the OF + PZQ and OF + Cur:Na_2_GA hamsters, there was less exploratory activity in the second trial relative to that of the first one, as measured by the duration and/or number of rearings of hamsters near a wall. These parameters were markedly or statistically significantly higher in the first trial of the test among the majority of the hamsters treated with a substance as compared to those of the CON group. Perhaps this is a manifestation of the elevated arousal of infected animals in response to drug administration and exposure to unfamiliar test conditions; this test is considered to be a mild stressor for them [45]. In contrast, after 30 days of the administration of substances, the arousal level returned to the baseline, meaning that they experienced habituation to the stressful conditions. Another distinctive feature of the infected animals compared to the CON hamsters was the following behavioral pattern: digging in corners of the field (number and duration), which was especially pronounced in groups OF + PZQ and OF + Cur. Animals in groups OF and OF + Cur:Na_2_GA did not differ from the CON hamsters in terms of the number and duration of corner digging in both cases. Regardless of what this pattern of behavior expresses (a panic response, anxiety, displacement activity, or something else), it is likely that PZQ and Cur, at these doses of administration, affect the brain by changing the behavior of hamsters because they [16,24,37,38] cross the blood–brain barrier. In this context, the effect of Cur:Na_2_GA on the brain is still neutral.

#### 3.2.2. Inspection of the Pancreatic Duct

**Figure 5:**
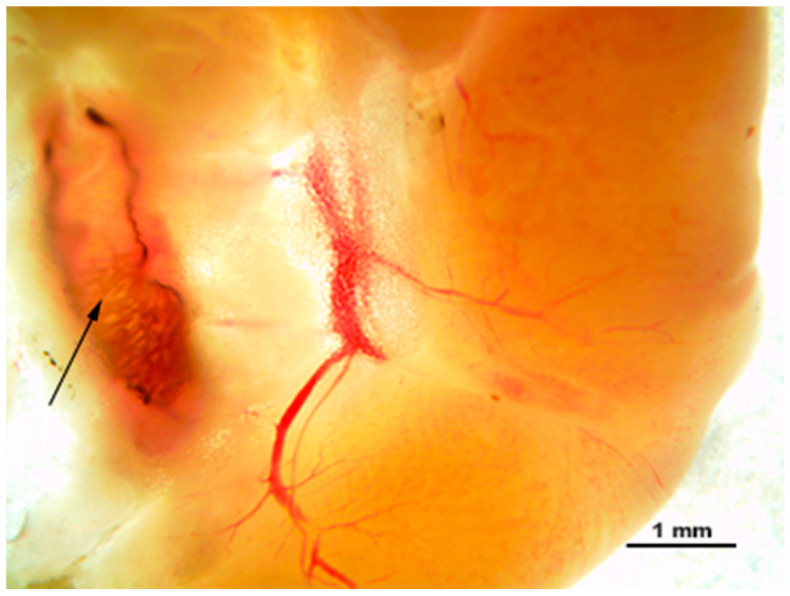
A native preparation (on a microscopy slide) of adult *O. felineus* (arrow) in the pancreas of a golden hamster (*Mesocricetus auratus*).

Among 19% of all infected animals, one adult *O. felineus* liver fluke was found in the pancreatic duct, which before flowing into the duodenum, merges with the common bile duct of the liver (Figure 5).

#### 3.2.3. The Livers of Golden Hamsters

**Figure 6:**
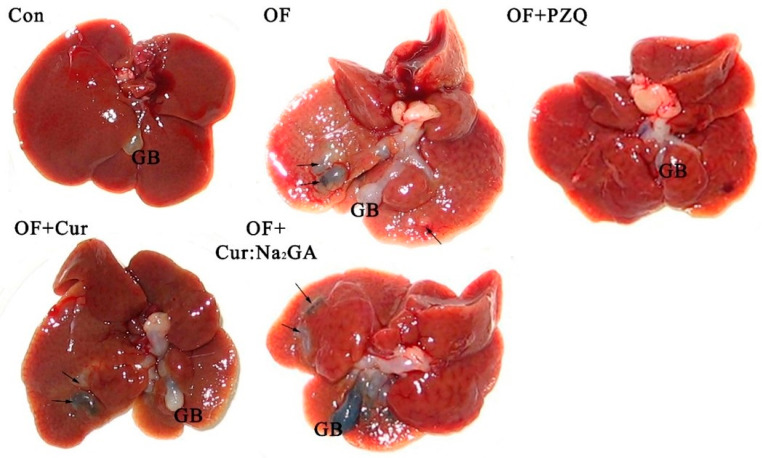
Macroscopic pictures of the visceral surface of the livers, gallbladders, and extrahepatic bile ducts in hamsters of various groups. GB: gallbladder. Animal groups’ names: Con: control, uninfected, untreated hamsters; OF: hamsters infected with *O. felineus*; OF + PZQ: hamsters infected with *O. felineus* and treated with a single administration of PZQ; OF + Cur: hamsters infected with *O. felineus* and treated with curcumin for 30 days; OF + Cur:Na_2_GA: hamsters infected with *O. felineus* and treated with the Cur:Na_2_GA complex for 30 days. The arrows point to subcapsular cholangiectasis filled with dark contents. The scale bar is 1 cm.

The autopsy of the CON animals revealed no pathological changes in the liver (Figure 6). In all other animals, the liver was proved to be enlarged. The extrahepatic bile ducts were tortuous, with cystic dilatations, and their walls were thickened and sclerosed. Cholangioectases on the visceral surface of the liver and gallbladder were found to be filled with black, gelatinous content. Thus, 30 days of the administration of the tested substances at 3 months after the initiation of the infection did not improve the livers’ state. Nevertheless, the livers appeared to be better in the hamsters treated with either PZQ or Cur as compared to the livers from groups OF + Cur:Na_2_GA and OF.

#### 3.2.4. Anthelmintic Effects of the Compounds

**Figure 7:**
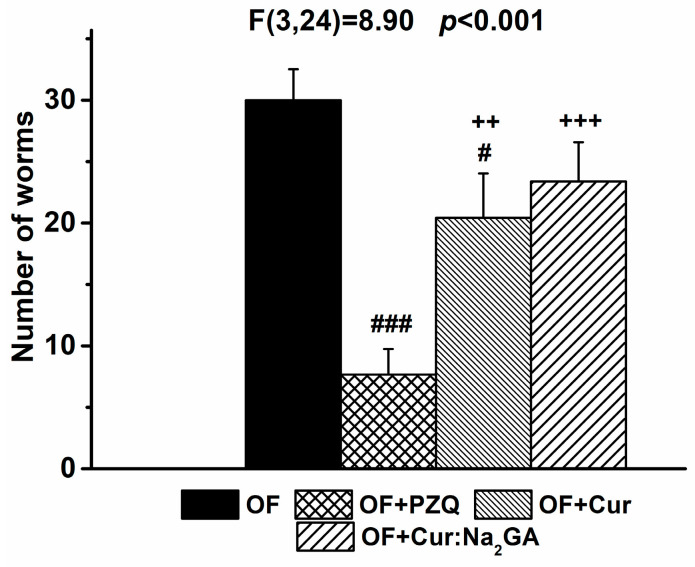
The number of helminths in the liver of hamsters of various groups. ^#^ *p* ≤ 0.05 and ^###^ *p* ≤ 0.001 as compared with group OF; **^++^** *p* ≤ 0.01 and **^+++^** *p* ≤ 0.001 as compared with group OF + PZQ (one-way ANOVA).

As shown in Figure 7, PZQ exerted the strongest anthelmintic effect, which reduced the infection rate in the liver by 74%.

A less-pronounced, but statistically significant, decrease in the number of parasites in the liver (by ~32%) occurred after the treatment with Cur was given. By contrast, after the administration of Cur:Na_2_GA, the hamsters did not differ from the OF hamsters in this parameter, possibly owing to the lower content of the active ingredient in it.

#### 3.2.5. Body Weights and Relative Liver and Spleen Weights

**Figure 8:**
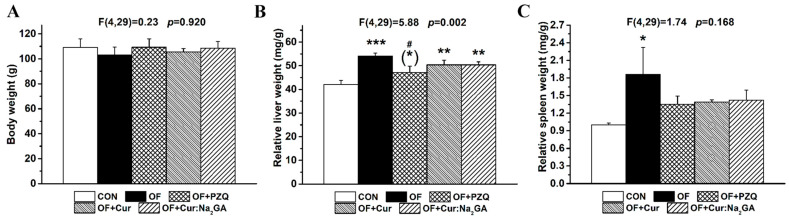
Body weight (**A**) and relative weights of the livers (**B**) and spleens (**C**) in the hamster groups. * *p* ≤ 0.05, ** *p* ≤ 0.01, *** *p* ≤ 0.001, and (*) 0.05 ≤ *p* ≤ 0.1 as compared with group CON; ^#^ *p* ≤ 0.05 as compared with group OF (one-way ANOVA).

At the end of the experiment, the hamster groups, which were initially randomized by body weight, did not differ in terms of body weight after the treatment with the tested substances (Figure 8A).

Four months after infection initiation, the OF hamsters had a significantly increased relative liver weight (Figure 8B). This parameter diminished after PZQ administration; the hamsters in this group differed from the CON hamsters only at the level of the trend (0.05 < *p* < 0.1), and they significantly differed from the OF hamsters (*p* < 0.05). The effects of Cur and Cur:Na_2_GA were equally weak, and the liver weights were still higher than they were in the CON animals. The infection of hamsters caused an increase in the relative weight of a peripheral organ of the immune system: the spleen (Figure 8C). Of note, Cur and Cur:Na_2_GA, just as PZQ did, reduced this parameter to the level of the CON hamsters.

#### 3.2.6. Biochemical Results

The hamsters’ infection with *O. felineus* helminths caused an increase in ALT activity by more than four-fold (Table 3).

The administration of PZQ, Cur, or Cur:Na_2_GA did not normalize this parameter. The AST activity level was found to be elevated in the infected hamsters after the administration of Cur:Na_2_GA (compared to those of both the CON hamsters and OF hamsters), but this was within the normal range for golden hamsters [25]. The animals in the five groups did not differ in terms of blood glucose level, except for a slight increase in the OF + PZQ animals, which, nevertheless, was within the normal range [25]. The administration of Cur produced a statistically significant decrease in the total amount of blood protein in the OF + Cur hamsters, and there was a more modest decrease in the OF and OF + PZQ hamsters. The administration of Cur:Na_2_GA helped maintain the amount of total protein at the levels seen in the CON hamsters. The biggest changes were registered in lipid metabolism. The infection elevated the cholesterol and triglyceride concentrations in the blood, but Cur, just as PZQ did, lowered the level of cholesterol to the control levels and was more effective than PZQ was at normalizing triglycerides. It is noteworthy that complex Cur:Na_2_GA, containing almost three times less Cur [31] and helped to maintain cholesterol and triglycerides near the control levels, although its influence on blood cholesterol was weaker than that of PZQ and Cur. Given that lipid metabolism in hamsters is similar to that of humans [46], a similar beneficial effect of curcuminoids can be expected to be found among humans with opisthorchiasis.

#### 3.2.7. Gene Expression Analysis in Liver Samples

**Figure 9:**
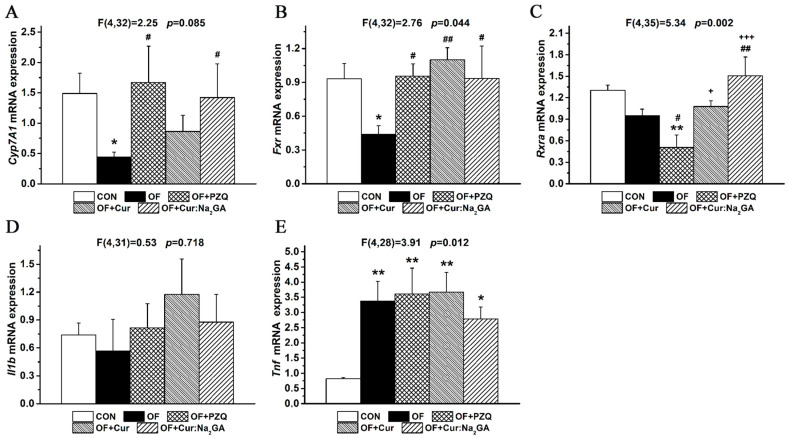
Relative expression levels of genes in the liver of hamsters from five groups (**A**–**E**). * *p* ≤ 0.05 and ** *p* ≤ 0.01 in comparison with group CON; ^#^ *p* ≤ 0.05 and ^##^ *p* ≤ 0.01 in comparison with group OF; **^+^** *p* ≤ 0.05 and **^+++^** *p* ≤ 0.001 in comparison with group OF + PZQ (one-way ANOVA).

In *O. felineus*-infected hamsters, the expression level of the key bile acid synthesis gene *Cyp7A1* turned out to be reduced (Figure 9A).

The administration of the tested substances, especially of PZQ and Cur:Na_2_GA, normalized the expression of this gene. In this analysis, the Cur:Na_2_GA complex, which contains less Cur per dose, had a more pronounced effect than free Cur did.

The expression of the gene of farnesoid X receptor, FXR, whose endogenous ligand in the liver is bile acids [47], was significantly lower in the OF hamsters (Figure 9B). Cur and Cur:Na_2_GA, just as PZQ did, increased the *Fxr* expression to the levels seen in the uninfected hamsters.

The expression level of the gene of retinoid X receptor, RXR, whose protein molecules form heterodimers with the FXR protein and participate in bile acid synthesis and cholesterol absorption [48], insignificantly declined after the initiation of *O. felineus* infection (*p* = 0.105; Figure 9C). PZQ administration, however, significantly inhibited the expression of this gene in the infected hamsters. In contrast to PZQ, the administration of Cur, and especially Cur:Na_2_GA, normalized the expression level of the *Rxr* gene.

The expression of genes that reflect inflammation (*Il1b* and *Tnf*) differently changed among the hamster groups. The groups did not differ in terms of the level of *Il1b* expression (Figure 9D). At the same time, *O. felineus* infection more than doubled the level of *Tnf* mRNA expression, which did not decrease after the treatments with PZQ, Cur, or Cur:Na_2_GA (Figure 9E).

#### 3.2.8. Histological Data

**Figure 10:**
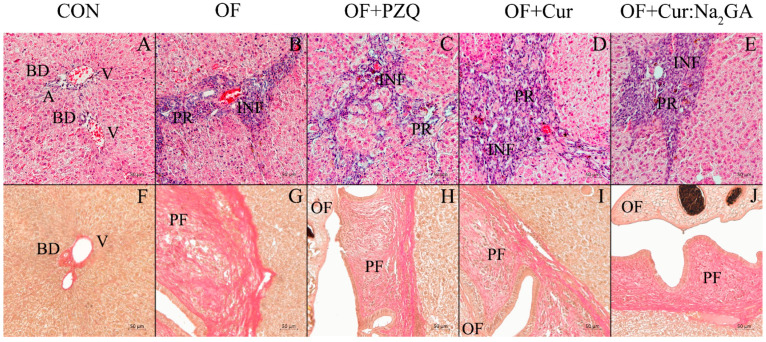
Representative histological samples of hematoxylin & eosin (**A**–**E**) and Van Gieson (**F**–**J**) staining of hamster liver slices. BD: bile duct; A: artery; V: vein; INF: cellular infiltrates; PF: periductal fibrosis; PR: bile duct proliferation; OF: *O. felineus*.

The histological assessment of liver samples from the control animals (CON) detected no pathological changes (Table 4, Figure 10A,F). The architectonics of the liver were normal; in the region of portal tracts, an artery, a vein, and a small bile duct were well visible; there were a few loci of aseptic inflammation and the weak proliferation of bile ducts, which is associated with age-related alterations in this organ. There were no fibrotic changes in the liver tissue.

In the OF hamsters, the proliferation of small bile ducts with cholangiofibrosis was registered, as was more pronounced periductal fibrosis in comparison with those of the other groups (Table 4, Figure 10B,G). Inflammatory infiltrates were present throughout the examined liver tissue and were predominantly of lymphocytic–monocytic origin, with a few neutrophils and eosinophils. After the treatment with PZQ, bile duct proliferation persisted, and inflammatory infiltration and cholangiofibrosis increased (Table 4, Figure 10C,H). In the hamsters treated with PZQ, the level of periductal fibrosis was half as pronounced as that in the OF hamsters. In the OF + Cur animals, small bile duct proliferation increased throughout the liver tissue and was the greatest compared to those of the other infected hamsters, whereas inflammatory infiltration declined and was localized predominantly to areas of small bile duct proliferation and periductal fibrosis (Table 4, Figure 10D,I). The severity of periductal fibrosis also diminished, but less markedly than that in the OF + PZQ animals. In the animals treated with the Cur:Na_2_GA complex, bile duct proliferation and cholangiofibrosis persisted, and these animals did not differ in terms of inflammation from the OF hamsters (Table 4, Figure 10E,J). A notable feature of the new complex was that it was better than free Cur was at preventing periductal fibrosis, and this benefit was comparable to that seen in the OF + PZQ group.

#### 3.2.9. Immunohistochemical Data

**Figure 11:**
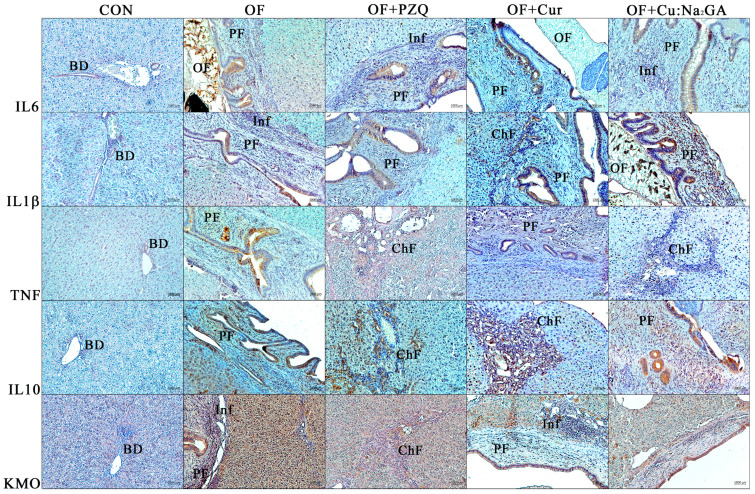
Immunohistochemical analysis of liver sections from five groups of hamsters. IL-6: interleukin 6; IL1β: interleukin 1 beta; TNF: tumor necrosis factor; IL-10: interleukin 10; KMO: kynurenine-3-monooxygenase; BD: bile duct; PF: periductal fibrosis; OF: *O. felineus*; Inf: cellular infiltrates; ChF: cholangiofibrosis.

As shown in Figure 11, IL-6- and IL-1β-positive cells were located predominantly in the epithelium of large and small bile ducts surrounded by fibrosis in all the groups of infected hamsters.

The effects of the administered drugs (PZQ, Cur, and Cur:Na_2_GA) on the magnitude of these parameters were not detectable visually. In the OF hamsters, TNF-positive staining was present both in hepatocytes of the liver and in the foci of inflammatory infiltration and persisted after the treatment with PZQ. The administration of Cur, but not Cur:Na_2_GA, attenuated this parameter; TNF-positive staining was present only in the cholangiocytes. Along with proinflammatory cytokines, the presence of anti-inflammatory cytokine, IL-10, was registered in the cholangiocytes of OF hamsters. It was also detected in the epithelium of the bile ducts surrounded by periductal fibrosis and in the animals treated with PZQ or Cur. In the OF + Cur:Na_2_GA animals, the level of IL-10-positive staining was the weakest. Via kynurenine-3-monooxygenase-positive staining, the groups could be ranked as follows: OF ≥ OF + PZQ > OF + Cur > OF + Cur:Na_2_GA. Moreover, this marker of inflammation was present both in the epithelium of the large and small bile ducts surrounded by fibrosis and in the hepatocytes of hamsters from groups OF and OF + PZQ. After the Cur treatment, and especially the Cur:Na_2_GA treatment, kynurenine-3-monooxygenase-positive staining was present only in the cholangiocytes.

## 4. Discussion

This study compares the effects of the hydrophobic polyphenolic substance, Cur, and of its water-soluble micellar complex, Cur:Na_2_GA (molar ratio 1:1), on trematode, *O. felineus,* as compared to the action of PZQ (the drug of choice for opisthorchiosis treatment).

Native and micellar Cur showed marked anthelmintic effects in vitro, especially on adult liver flukes. The impacts on NEMs of *O. felineus* were similar between Cur and PZQ, supporting the potential use of Cur as an anti-opisthorchiosis agent. The effect of Cur:Na_2_GA on NEMs was weaker, probably due to the lower content of free Cur in it.

In the in vivo experiment, a good anthelmintic effect of Cur was documented here after the administration to *O. felineus*-infected animals for 30 days, although the benefit was less pronounced than that of PZQ. This finding contradicts the data obtained regarding *O. viverrini*-infected hamsters; no significant difference in the number of eggs per gram of feces was found between the animals of a normal diet group and a 1% Cur-consuming group after 30 days of treatment [17]. We believe that the administration of Cur to *O. felineus*-infected hamsters for longer (3 months or more) is necessary to achieve a more pronounced anthelmintic effect. The absence of anthelmintic activity in the Cur:Na_2_GA complex is probably due to the threefold lower weight content of the active ingredient, despite there being a ~19-fold increase in bioavailability as compared to that of free Cur [31].

Furthermore, Cur and Cur:Na_2_GA, just as PZQ did, reduced the relative weight of the spleen, which was found to be elevated in the OF hamsters, possibly pointing to the normalization of functions of this organ in the host’s immune system. Visually, all the animals tolerated the administered drugs well because they steadily gained body weight and did not differ in terms of this parameter at the end of the experiment. At the same time, the relative weight of the livers remained elevated, which, apparently, is due to the presence of helminths in it.

Adult *O. felineus* liver flukes were found within the pancreatic duct in a large proportion of the infected hamsters, including after the administration of various substances. It is known that the presence of helminths in the pancreas leads to the same changes as those in bile ducts of the liver and contributes to the impairment of the secretory function of the pancreas, owing to chronic proliferative cholangitis and pancreatic canaliculitis, which is accompanied by fibrosis of various degrees [49]. In patients with opisthorchiosis caused by *O. felineus*, pancreatitis is a common complication [50]. The reason is that getting rid of helminths is extremely difficult in case of parasitism in the pancreas.

Regardless of the absence of anthelmintic activity in Cur:Na_2_GA, we noticed its positive effects on the host’s state, which were sometimes even more pronounced than those of free Cur. For instance, Cur:Na_2_GA, just as Cur and PZQ did, helped to maintain normal lipid metabolism in the parasite’s host by attenuating the elevation of blood cholesterol and triglyceride levels. A similar hypocholesterolemic effect of Cur, which is attributed to the inhibition of intestinal absorption of cholesterol, has been demonstrated in pathogen-free hamsters that consumed high-fat food supplemented with Cur (0.05–0.1% *w*/*w*) for 3 months [51,52]. The authors demonstrated that Cur promotes a rise in the total amount of bile acids, which is accompanied by a higher expression level of cholesterol-7α-hydroxylase protein and a higher *CYP7A1* mRNA expression level in the liver [52]. During these processes, the involvement of farnesoid X receptor (FXR) in the small intestine cannot be ruled out, which has been shown to participate in lipoprotein metabolism [53]. The activation of these receptors by FXR agonists is known to lower the plasma triglyceride levels in mice and rats [53,54].

Of the 17 enzymes in the classic pathway of the biosynthesis of bile acids from cholesterol, cholesterol 7α-hydroxylase is regarded as the key enzyme [55,56]. In our experiments, the *CYP7A1* mRNA expression level was low in the *O. felineus*-infected hamsters, and this aberration was accompanied by higher blood cholesterol and triglyceride levels. Under these conditions, the administration of Cur:Na_2_GA, similarly to PZQ, enhanced the expression of *CYP7A1* in the liver, as well as improving lipid metabolism. Similarly, the under-expression of the *CYP7A1* gene has been shown in hamsters infected with another member of the Opisthorchiidae family: *O. viverrini* [26]. In those experiments, however, only the combined administration of nano-encapsulated Cur with PZQ significantly increased this parameter during chronic opisthorchiosis.

The expression of the *CYP7A1* gene is believed to be controlled via negative feedback by ligand-activated transcription factor FXR, which is a member of the nuclear receptor superfamily [55,57]. FXR, together with retinoid X receptor (RXR), form a heterodimer that inhibits 7-α hydroxylase, and thereby, blocks the conversion of cholesterol into bile acids. Nonetheless, there is not always a direct relation between the activation of *Fxr* gene expression and the suppression of *CYP7A1*, as demonstrated in hamsters that were infected with *O. viverrini* for 3 months [57]. In our experiments, the under-expression of the *CYP7A1* gene in OF hamsters was concurrent with the downregulation of the *Fxr* gene and unchanged *Rxr* expression level.

It is worth drawing the readers’ attention to the pronounced pathological structural changes in the liver of *O. felineus*-infected animals: a combination of fibrosis and cholangiofibrosis with extensive foci of inflammatory infiltration. After the treatment with the tested substances, cholangiofibrosis persisted, while periductal fibrosis was markedly inhibited by Cur:Na_2_GA and the level of it was comparable to that in animals treated with PZQ and less than that of periductal fibrosis in the hamsters treated with free Cur. Free Cur, however, unlike the other compounds, was more effective at reducing inflammatory infiltration. Another distinctive feature of free Cur was the induction of proliferative activity in the liver, which was an increase in the number of bile ducts. There are conflicting data on the proliferative properties of Cur. For instance, in a rat model of experimental hepatectomy, even 7 days of the administration of 100 mg/kg Cur increases the proliferative index by more than 20-fold, as estimated in terms of proliferating cell nuclear antigen expression [58]. On the contrary, in experiments involving diethylnitrosamine-induced liver inflammation, as an example, the expression of PCNA diminishes after Cur was administered to rats [59].

Severe inflammation in the liver was also confirmed via immunohistochemical analysis: the extent of kynurenine-3-monooxygenase (KMO)-positive staining was the most pronounced in OF hamsters among all the groups and was accompanied by the upregulation of proinflammatory cytokines (IL-6, IL-1β, and TNF). The relationship between these parameters has been reported by other researchers: in cultured rat pancreatic islet cells, the addition of IL-1β raises the protein expression level of KMO [60]. Kynurenine-3-monooxygenase (just as indoleamine-2,3-dioxygenase 1, IDO1, does) is a rate-limiting enzyme of the kynurenine pathway of tryptophan metabolism in the body and is activated in response to immunostimulation caused by external factors (e.g., infection, inflammation, dietary changes, various stressors, and peripheral exposure to lipopolysaccharide) [61,62]. In addition to participation in inflammatory processes, KMO is reported to be over-expressed in a malignant phenotype of human hepatocellular carcinoma [63]. Enhanced KMO staining was found in our work, not only in the cholangiocytes, but also in the liver hepatocytes of OF hamsters, which reflects severe inflammation. As a consequence of this inflammation in the livers of OF hamsters, the synthesis of bile acids is disturbed, as is the supply of bile acids, cholesterol, phospholipids, and small amounts of bile pigments and protein constituents of bile in the gall bladder for subsequent secretion into the small intestine, with 95% reabsorption back into the liver. Cholestasis is due to substantial concentrations of hemozoin and mucus in the ducts and gallbladders of infected animals, which contribute to lower rates of synthesis of bile and disturbances of bile transport processes and bile reabsorption in the intestine. A correlation between the severity of the symptoms and cholestasis is observed in 50% of *O. felineus*-infected people [5]. The impaired tonic and motor function of bile ducts and of the gall bladder and the progression of cholestasis play a leading role in the pathogenesis of the chronic stage of opisthorchiosis [64]. In this regard, among humans, there is evidence of elevated levels of serum enzymes alkaline phosphatase and transaminase and low plasma levels of albumin, which are attributed to hepatic cirrhosis and cholestasis [65]. In the OF hamsters in our experiments, the ALT activity level was also found to be increased. The treatment with Cur did not normalize this parameter. The treatment with PZQ markedly attenuated the activity level of ALT, possibly owing to PZQ’s more effective anthelmintic action. It has been shown that during the infection of hamsters with a two-fold smaller dose of *O. viverrini* (50 metacercaria/hamster versus 100 metacercaria/hamster in our experiments), consuming food containing 1% Cur for 30 days reduces the ALT activity level, albeit not to the control values [17].

It is possible that during inflammation induced by *O. felineus*, the mechanisms regulating the classic synthesis of bile acids are disconnected, and an alternative pathway is switched on due to the participation of mitochondrial sterol 27-hydroxylase (CYP27A1). The launch of this pathway in pathologies of the liver has been theorized by some authors [56]. This regulation is related to c-Jun N-terminal kinase (JNK) and does not depend on FXR [53,66]. In addition, other studies have shown that tumor necrosis factor (TNF), which triggers the JNK signaling pathway, can repress the mRNA expression of *CYP7A1* [67]. In this regard, it is worth mentioning the *Tnf* gene over-expression observed in OF hamsters in our work; this over-expression did not change after the treatment with the tested substances was administered, thereby confirming the persistence of severe inflammation of the liver. Immunohistochemically, we also documented a high level of the TNF protein in the OF and OF + PZQ hamsters. Less intense TNF staining was seen after the treatment with Cur, but not Cur:Na_2_GA. Presumably, the mechanism of action of Cur entails the inhibition of transcription factor NF-κB, as demonstrated by other researchers; Cur blocks both the expression and signaling of TNF, and Cur’s anti-inflammatory effects are mediated via the suppression of transcription factor, NF-κB [14].

Another indicator of the severe inflammation of the livers of *O. felineus*-infected hamsters is the considerable amount of IL-6- and IL-1β-positive staining in the epithelium of the large and small bile ducts surrounded by fibrosis, simultaneously with IL-10-positive cells; the expression of these cytokines persisted after the treatment with the tested substances. At the same time, in the liver parenchyma, no differences were found in the level of expression of *Il1b* mRNA among the five groups of hamsters, in contrast to *Tnf* mRNA. There is evidence from Western blot analysis, indicating the persistent upregulation of the TNF protein during the progression of experimental opisthorchiosis for up to 1.5 years [68]. Together with the results of our study, these data imply a key role of this proinflammatory cytokine in the maintenance of severe inflammation during *O. felineus* infection. The exclusivity of TNF, in this regard, is evidenced by the high protein and mRNA expression levels; furthermore, the treatment with Cur, just as that with PZQ, under these conditions was not sufficient to reduce this severe inflammation.

It is also worth mentioning the ability of curcuminoids to act on some central mechanisms of behavior regulation because these compounds can pass through the blood–brain barrier, which is similar to the ability of PZQ [16,24,37,38]. In the present study, when hamsters were tested in the open field test, an increase in exploratory behavior (rearing duration and/or the number of rearings of hamsters) was registered in the first hours after the first administration of the tested substances; this behavior normalized after 30 day of administration of the substances. Whether this is a panic-driven avoidance response or elevated exploratory activity remains to be determined. We also do not rule out the effect of arousal in infected hamsters in the first hours after the administration of the substances or a solvent. Nevertheless, specific effects of PZQ, Cur, and Cur:Na_2_GA are also possible because in OF hamsters, indicators of exploratory behavior were the same in both trials of the test.

## 5. Conclusions

Under in vitro and in vivo conditions, the effects of Cur and of its new water-soluble complex, Cur:Na_2_GA (1:1), were compared with each other and with the actions of a clinically used anthelmintic, PZQ. The anthelmintic efficacy of Cur against adult *O. felineus* helminths is shown for the first time. Despite the lower content of the active ingredient, the micellar Cur:Na_2_GA complex, just as PZQ and free Cur did, improved some physiological and biochemical parameters and the expression of genes associated with bile formation and reduced the expression of proteins induced by inflammation in the livers of *O. felineus*-infected hamsters. This indicates that this complex holds promise as a remedy against the negative consequences of trematode infection for hosts’ health. Thus, Cur and Cur:Na_2_GA have beneficial properties for the treatment of opisthorchiosis in humans. Future research will show how these findings can be applied in practice.

## Figures and Tables

**Table 1:** IC_50_ values of the tested substances.

	NEMs *	Adult Flukes
**Na_2_GA**	30,400 μg/mL	1236.470 μg/mL
**PZQ**	0.587 μg/mL	0.208 μg/mL
**Cur**	0.611 μg/mL	0.500 μg/mL
**Cur:Na_2_GA (1:1)**	31.720 μg/mL	3.007 μg/mL

* Newly excysted metacercariae.

**Table 2:** Behavior of hamsters from five groups in the open field test.

Trials	Behavioral Parameters	Experimental Groups				
		CON	OF	OF + PZQ	OF + Cur	OF + Cur:Na_2_GA
1	Orienting reaction time, s	7.2 ± 3.32	7.1 ± 1.81	9.2 ± 1.52	11.6 ± 4.15	10.6 ± 4.34
2	Orienting reaction time, s	12.4 ± 2.62	11.1 ± 1.81	13.7 ± 3.24	12.2 ± 4.21	9.9 ± 3.95
1	Number of crossed squares	45.2 ± 10.13	69.7 ± 10.18	45.0 ± 8.08	64.0 ± 12.34	78.3 ± 11.18 (*) **+**
2	Number of crossed squares	47.7 ± 6.70	45.8 ± 7.84 (**a**)	24.2 ± 5.07 * (**#**) (**a**)	43.1 ± 10.82	40.8 ± 5.80 **+ a**
1	Number of rearings	3.4 ± 1.08	8.8 ± 1.91 *	8.4 ± 1.70 (*)	12.1 ± 2.70 *	11.2 ± 2.46 *
2	Number of rearings	5.3 ± 2.29	7.7 ± 2.45	1.7 ± 0.42 **# aa**	7.9 ± 1.91 **+**	5.1 ± 2.58 (**a**)
1	Rearing duration, s	9.6 ± 2.69	16.2 ± 4.11	18.8 ± 4.76	26.1 ± 4.76 *	21.7 ± 4.42 *
2	Rearing duration, s	14.9 ± 6.38	13.6 ± 4.07	4.6 ± 1.65 **a**	19.8 ± 3.7 **+**	13.3 ± 7.12
1	Number of corner diggings	0.7 ± 0.49	2.3 ± 1.02	3.6 ± 1.03 (*)	3.4 ± 1.18 (*)	0.9 ± 0.42 (**+**)
2	Number of corner diggings	1.1 ± 0.66	3.2 ± 1.44	5.5 ± 1.79 *	4.4 ± 1.42 (*)	2.3 ± 0.84
1	Corner digging duration, s	1.2 ± 1.01	12.0 ± 9.10	23.7 ± 9.39 (*)	17.8 ± 7.42 (*)	4.4 ± 2.71
2	Corner digging duration, s	2.6 ± 1.77	16.2 ± 8.50	48.9 ± 18.39 * (**#**)	17.8 ± 5.29 *	19.1 ± 8.77
1	Time near central empty Falcon® tube, s	2.3 ± 0.59	1.7 ± 0.36	2.3 ± 1.10	0.7 ± 0.39 (*) **#** (**+**)	1.7 ± 0.48
2	Time near central empty Falcon® tube, s	2.9 ± 0.75	2.5 ± 0.43	2.9 ± 0.66	1.9 ± 0.89	4.6 ± 1.57
1	Time near left empty Falcon® tube, s	3.5 ± 1.02	3.0 ± 0.95	3.8 ± 1.19	1.9 ± 0.50	3.2 ± 0.46 (**c**)
2	Time near Falcon® tube containing food pellets, s	14.9 ± 2.01 **aa**	22.6 ± 5.06 **aa**	16.9 ± 4.84 **a**	20.8 ± 6.16 (**a**)	28.2 ± 4.32 * **aaa**
1	Time near right empty Falcon® tube, s	4.7 ± 1.92	6.0 ± 1.92	6.5 ± 2.90	2.7 ± 0.89 (**#**)	4.8 ± 1.20
2	Time near Falcon® tube with valerian, s	3.4 ± 1.05	4.1 ± 2.24 (**a**)	8.0 ± 3.32	6.1 ± 2.03	10.7 ± 3.26

Trial 1: determination of behavioral parameters at 3–6 h after substance administration; Trial 2: determination of behavioral parameters after 30 days of the administration of substances. **Comparisons within rows**: * *p* ≤ 0.05 and ^(^*^)^ 0.05 ≤ *p* ≤ 0.1 in comparison with group CON; **^#^** *p* ≤ 0.05 and ^(**#**)^ 0.05 ≤ *p* ≤ 0.1 in comparison with group OF; **^+^** *p* ≤ 0.05 and ^(**+**)^ 0.05 ≤ *p* ≤ 0.1 in comparison with group OF + PZQ; ^(**c**)^ 0.05 ≤ *p* ≤ 0.1 in comparison with group OF + Cur, according to the Mann–Whitney *U* test. **Comparisons within columns**: **^a^** *p* ≤ 0.05, **^aa^** *p* ≤ 0.01, **^aaa^** *p* ≤ 0.001, and ^(**a**)^ 0.05 ≤ *p* ≤ 0.1 in comparison with Trial 1, according to the Mann–Whitney *U* test.

**Table 3:** Changes in blood biochemical parameters in hamsters in five groups.

Parameters	Normal Range Ref. [25]	CON	OF	OF + PZQ	OF + Cur	OF + Cur:Na_2_GA	ANOVA
ALT, U/L	22–128	57.3 ± 5.53	242.0 ± 39.58 ***	179.3 ± 50.4 *	263.7 ± 41.96 ***	199.3 ± 26.7 **	**F_(4,29)_ = 4.62** ***p* = 0.005**
AST, U/L	20–150	107.0 ± 23.35	109.4 ± 10.40	117.0 ± 13.52	136.3 ± 10.24	150.0 ± 12.93 * ^#^	F_(4,29)_ = 1.79 *p* = 0.158
GLU, mmol/L	3.3–8.3	4.2 ± 0.18	4.8 ± 0.65	5.3 ± 0.22 (*)	5.0 ± 0.41	4.5 ± 0.37	F_(4,29)_ = 1.03 *p* = 0.407
TP, g/L	45–47	88.3 ± 3.45	80.7 ± 3.19 (*)	79.9 ± 3.21 (*)	74.4 ± 2.01 **	81.4 ± 3.26	F_(4,29)_ = 2.46 *p* = 0.067
CHOL, mmol/L	1.4–4.7	4.0 ± 0.23	5.2 ± 0.31 *	3.7 ± 0.25 ^##^	3.9 ± 0.52 ^##^	4.8 ± 0.27 ^+^ (^c^)	**F_(4,29)_ = 3.54** ***p* = 0.018**
TG, mmol/L	0.81–2.56	1.9 ± 0.17	2.6 ± 0.18 *	2.0 ± 0.36 (^#^)	1.8 ± 0.14 ^#^	2.3 ± 0.14	F_(4,29)_ = 2.18 *p* = 0.096

ALT: alanine aminotransferase; AST: aspartate aminotransferase; GLU: glucose; TP: total protein; CHOL: total cholesterol; TG: triglycerides. Normal range: serum biochemistry parameters of Syrian golden hamsters (*Mesocricetus auratus*), as published by Chanakan Jantawong with co-authors [25]. * *p* ≤ 0.05, ** *p* ≤ 0.01, *** *p* ≤ 0.001, and (*) 0.05 ≤ *p* ≤ 0.1 as compared with group CON; ^#^ *p* ≤ 0.05, ^##^ *p* ≤ 0.01 and ^(#)^ 0.05 ≤ *p* ≤ 0.1 as compared with group OF; **^+^** *p* ≤ 0.05 as compared with group OF + PZQ; ^(c)^ 0.05 ≤ *p* ≤ 0.1 as compared with group OF + Cur (one-way ANOVA).

**Table 4:** A comparison of histological characteristics of the liver among the five groups of hamsters.

Parameters	CON	OF	OF + PZQ	OF + Cur	OF + Cur:Na_2_GA	ANOVA
Proliferation small bile duct, %	0.93 ± 0.31	17.17 ± 4.59 *	15.57 ± 5.12 (*)	22.44 ± 8.44 **	17.65 ± 2.28 *	F_(4,21)_ = 2.60 *p* = 0.065
Cellular infiltrations, %	0.08 ± 0.06	6.11 ± 1.05 (*)	8.40 ± 4.28 *	4.00 ± 0.99	5.95 ± 1.92 (*)	F_(4,20)_ = 1.73 *p* = 0.183
Cholangiofibrosis, %	0.00 ± 0.00	16.80 ± 7.61 (*)	22.77 ± 9.39 *	21.47 ± 6.78 *	21.27 ± 7.35 *	F_(4,21)_ = 1.71 *p* = 0.185
Periductal fibrosis, %	0.00 ± 0.00	48.41 ± 2.28 ***	24.47 ± 3.27 *** ###	34.59 ± 7.21 *** #	23.68 ± 6.35 ** ###	**F_(4,21)_ = 16.06** ***p* < 0.001**

* *p* ≤ 0.05, ** *p* ≤ 0.01, *** *p* ≤ 0.001, and (*) 0.05 ≤ *p* ≤ 0.1 in comparison with group CON; **^#^** *p* < 0.05 and **^###^** *p* ≤ 0.001 relative to group OF (one-way ANOVA).

## Data Availability

The data presented in this study are available on request from the corresponding author.
